# CAN YOU HEAR ME? A MEDICAL STUDENT-LED QUALITY IMPROVEMENT INITIATIVE FOR HOSPITALIZED PATIENTS WITH HEARING LOSS

**DOI:** 10.1093/geroni/igac059.2279

**Published:** 2022-12-20

**Authors:** Stephanie Rennke, Rachel Warren, Wynton Sims, Shreya Menon, Sasha Binford, Megan Rathfon, Margaret Wallhagen

**Affiliations:** University of California, San Francisco, San Francisco, California, United States; University of California, San Francisco, San Francisco, California, United States; University of California, San Francisco, San Francisco, California, United States; University of California, San Francisco, San Francisco, California, United States; University of California, San Francisco, San Francisco, California, United States; University of California, San Francisco, San Francisco, California, United States; University of California, San Francisco, San Francisco, California, United States

## Abstract

In acute care settings hearing loss (HL) is associated with impaired patient-provider communication, increased length of stay, and increased mortality. COVID-19 has exacerbated this problem with the widespread use of masks and eye shields which muffle speech and prevent lip reading. Our 800-bed tertiary care medical center lacked a standardized approach to identify patients with HL and address communication barriers. Three first year medical students spearheaded the initiative as part of a health systems improvement curriculum with support from a faculty coach and a faculty researcher. From September 2020 to October 2021, the students met with stakeholders, leadership and champions and unit staff, identified the current state and completed a gap analysis through interviews, surveys, and direct observation. The pilot in May 2021 included two week-long PDSA cycles on one hospital unit to: 1) screen patients aged 65 and older using the validated 10-item HHIE-S questionnaire, 2) implement an education and awareness campaign with bedside signage, posters, and conferences and 3) provide a personal amplifier (purchased in bulk by the medical center) with verbal and written instructions. A total of 29 patients screened positive and were given personal amplifiers. Post-pilot interviews reported increased provider awareness and knowledge around best communication practices. Patients and staff reported limited amplifier use due to poor sound quality, small dials, poorly fitting ear buds and a short battery life. Based on these results the team recommended discontinuing the personal amplifiers and identifying other communication tools including higher quality personal amplifiers and speech to text applications.

